# AI and Digital Tools in Dermatology: Addressing Access and Misinformation

**DOI:** 10.2196/79044

**Published:** 2026-02-20

**Authors:** Dominique du Crest, Monisha Madhumita, Wendemagegn Enbiale, Jose Antonio Ruiz Postigo, Josep Malvehy, Shannon Wongvibulsin, Somesh Gupta, Harald Kittler, Charbel Skayem, Anjali Mahto, Adewole S Adamson, Jules B Lipoff, Art Papier, Hugues Cartier, Sébastien Garson, Esther Freeman

**Affiliations:** 1 SkinAid SAS, 50 Rue Danton, Levallois Perret, 92300, France, 33 620715128; 2 Department of Dermatology, Saveetha Medical College, Chennai, India; 3 College of Medicine and Health Sciences, Bahir Dar University, Bahir Dar, Ethiopia; 4 World Health Organization, Geneva, Switzerland; 5 Centro de Investigación Biomédica en Red de Enfermedades Raras Instituto de Salud Carlos III, Barcelona, Spain; 6 Division of Dermatology, Department of Medicine, David Geffen School of Medicine, University of California, Los Angeles, CA, United States; 7 All India Institute of Medical Sciences, New Delhi, New Delhi, India; 8 Department of Dermatology, Medical University of Vienna, Vienna, Austria; 9 Hôpital Ambroise-Paré, Boulogne Billancourt, France; 10 Self London, London, United Kingdom; 11 University of Texas at Austin Dell Medical School, Austin, TX, United States; 12 Department of Dermatology, Lewis Katz School of Medicine, Temple University, Philadelphia, PA, United States; 13 Department of Dermatology, University of Rochester College of Medicine, Rochester, NY, United States; 14 Saint-Jean Clinic of Dermatology, Arras, France; 15 SELARL Dr GARSON, Senlis, France; 16 Massachusetts General Hospital, Department of Dermatology, Boston, MA, United States

**Keywords:** digital dermatology, AI in health care, global health, teledermatology, artificial intelligence, AI, large language model, LLM, health tech, skin, dermatology, digital health, misinformation, ethical AI, radical dermatology, social media

## Abstract

Digital dermatology, which is defined as the use of digital technologies that leverage individual- and population-level skin data to improve the diagnosis, treatment, and prevention of skin diseases, has emerged as a critical frontier for bridging persistent gaps in dermatologic care. This transformation holds particular promise for addressing long-standing inequities linked to geography, income, and skin type. According to the Global Burden of Disease 2023 study, skin and subcutaneous diseases remain among the most prevalent global health conditions, contributing substantially to disability-adjusted life years. Digital tools (including teledermatology, artificial intelligence [AI], and large language models) offer new ways to extend diagnosis, education, and patient empowerment to historically underserved populations. However, these same innovations risk amplifying disparities if they are not designed and deployed intentionally. Algorithmic bias, uneven digital access, and the absence of culturally responsive models can undermine progress. In this conceptual and narrative review, we draw on expert dialogues and illustrative literature, including multistakeholder exchanges at the *Skin and Digital Summit (2023-2025*) and related global forums, to examine how digital dermatology can promote equitable skin health. We focus on 3 interlinked priorities: expanding access through scalable digital platforms, ensuring AI fairness via comprehensive and diverse datasets, and countering dermatological misinformation. Central to the latter is a bot concept described here as a dynamic cycle that analyzes scientific literature; ranks evidence; translates complex research into clear language; and delivers trustworthy, personalized guidance to both consumers and clinicians. By embedding expert oversight and evidence prioritization, such tools can ensure that accurate, actionable information reaches users at the speed and scale of the internet. Drawing on case studies (including lessons from the World Health Organization’s AI skin health app) and insights from the Skin and Digital Summit*,* we highlight both the transformative potential and the ethical complexities of these digital solutions. To navigate this evolving landscape, we propose the concept of radical dermatology**,** which confronts the reality that big tech is reshaping skin health whether we like it or not and insists that dermatologists and stakeholders lead the transformation through bold collaboration and unwavering clinical relevance.

## Introduction

Globally, skin and subcutaneous diseases are among the most common health conditions. In 2019 alone, there were an estimated 4.86 billion new cases globally, with fungal (34%) and bacterial (23%) infections making up the majority of cases [1]. Skin conditions accounted for nearly 43 million disability-adjusted life years, a burden that is both physically and psychosocially disabling [1].

Despite this high burden, dermatologic care remains inaccessible for many. An estimated 70% of people living with chronic skin diseases, including psoriasis and rosacea, do not receive professional medical care, largely due to limited dermatology workforce capacity, cost barriers, geographic distance, and sociocultural factors in care delivery [2]. These barriers affect both low-resource settings and underserved communities in low-, middle-, and high-income countries [3].

At the same time, rapid smartphone penetration, the maturation of generative artificial intelligence (AI) tools, and the World Health Organization’s (WHO) prioritization of skin health have created an unprecedented window for digital solutions. Digital dermatology refers to the application of digital technologies and skin-related data (including telemedicine, mobile health [mHealth], AI, and digital imaging) to improve the diagnosis, treatment, and prevention of skin diseases. While it offers opportunities to bridge persistent gaps in access to care, challenges such as bias in algorithms, limited infrastructure, and variable digital literacy must be addressed through deliberate equity-focused design and governance. Unlike traditional models that rely on in-person specialist visits concentrated in urban centers, digital dermatology can decentralize expertise, shorten wait times, enable earlier diagnosis, extend specialist reach, and support patient self-management across geographies [4]. However, equity is not guaranteed. If bias goes unaddressed, if infrastructure remains weak, or if digital literacy lags, digital dermatology may deepen the very divides it seeks to close. In the following sections, we highlight strategies to counter these risks and to align innovation with equity.

This conceptual and narrative review synthesizes expert insights, stakeholder dialogue, and illustrative case examples to explore equity-centered strategies in digital dermatology. Input was drawn from international experts through 4 panels and 3 roundtables at the Skin and Digital Summit held virtually (IMCAS, December 2024) and in Paris (IMCAS, January 2025) [5]. Data were captured through structured notes and partial transcripts and then analyzed using inductive thematic coding by 2 reviewers, with disagreements resolved by consensus and findings organized through framework synthesis [6,7].

As discussions occurred in public professional forums, institutional review board approval was not required. Any direct speaker quotations were deidentified or quoted with permission, and related files were stored securely with restricted access, in compliance with data protection best practices.

To complement these expert perspectives, we conducted a targeted literature review (PubMed, Scopus, and Web of Science; English-language studies), including studies on equity, implementation, and ethics in digital dermatology and related fields, thereby reducing selection bias.

We discuss the WHO-neglected tropical disease mobile app as an example of ongoing implementation efforts and insights from global experts with references, where applicable. These examples were selected to highlight emerging real-world dynamics that are not yet captured in peer-reviewed literature. A narrative approach was used to thematically analyze insights across the digital dermatology continuum involving access to dermatologic care through digital innovation, datasets in dermatology, and combating skin-related misinformation.

## Expanding Access to Dermatologic Care Through Digital Innovation

**Figure 1.**
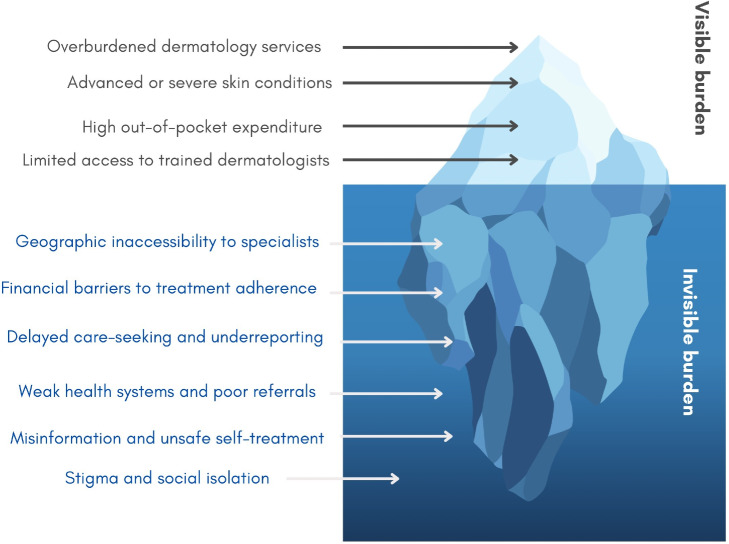
The iceberg phenomenon of dermatologic care disparities.

Despite the global burden of skin disease, dermatologic care remains underprioritized. Structural barriers, workforce shortages, and high out-of-pocket costs continue to limit access, especially in underserved and rural areas (Figure 1). These challenges are particularly acute for skin of color and populations in low- and middle-income countries [8,9].

Digital health innovations, including teledermatology, AI-assisted diagnostics, and mHealth apps, are increasingly recognized as promising tools to improve dermatology service delivery and care for all [10]. Given its reliance on visual diagnosis, dermatology makes it more amenable to remote care compared with many other specialties. High-quality clinical images and contextual data can enable remote triage, diagnosis, and follow-up, offering an opportunity to decentralize expertise [11,12].

One area is the proliferation of consumer-facing dermatology apps that offer mole monitoring, acne advice, and other self-assessment tools for timely triage and basic education, particularly in contexts where specialist access is limited. A 2024 review identified more than 900 such apps, including 41 with AI capabilities, and found their performance to be variable [13]. A 2021 analysis also showed sensitivity for melanoma to be highly variable across top apps, often with little dermatologist oversight or regulatory validation [14].

Alongside commercial tools, institutional efforts are advancing [15]. In 2024, the WHO piloted an AI-assisted mobile app across 5 Kenyan counties to help frontline workers identify 13 neglected tropical diseases and 24 common skin conditions. Forty Ministry of Health workers captured 605 patient images, which were reviewed by dermatologists. Although formal peer-reviewed accuracy data are pending, preliminary findings indicate more than 80% diagnostic agreement and strong usability for triage purposes rather than as a diagnostic replacement. Importantly, the app includes offline functionality and multilingual support and design features critical for resource-limited contexts [16,17]. However, several infrastructural and regulatory barriers persist as challenges, including unstable networks, device costs, limited digital literacy, and fragmented data governance frameworks that constrain uptake [18]. In some regions, legal restrictions on data sharing further complicate deployment. Addressing these challenges will require coordinated public-private partnerships and context-specific policy support.

Additionally, we must acknowledge that although digital dermatology tools can improve diagnosis and education, their impact is limited if patients cannot access the medications they are prescribed. In underserved areas, medication shortages, high costs, and weak supply chains often prevent timely treatment. Closing gaps in dermatologic care requires strategic, multisector partnerships to ensure that digital solutions are not just short-term pilot projects, but long-lasting, locally led interventions supported by active partnerships with big tech and big pharma [19]. To strengthen implementation, policy levers such as teledermatology reimbursement and data protection standards must be embedded within digital health initiatives. Such initiatives must work in tandem with public health systems, with continued monitoring of program success (such as shorter time to treatment after digital triage), through demand forecasting, community health worker training, and pharmacy partnerships for sustained equitable access to both diagnosis and treatment [20].

## Datasets: The Key to Unlocking AI Potential in Dermatology

Complementing access-focused interventions, the success of AI in digital dermatology hinges on one critical factor: data. Dermatologic AI systems predominantly rely on visual data, including clinical photographs and dermatoscopy images, for training and validation [21]. However, the lack of large, diverse, and representative datasets poses a major limitation [22]. Most existing datasets overrepresent light-skinned patients from high-income settings and focus heavily on conditions such as melanoma [23]. Even newer collections, such as the Diverse Dermatology Images dataset, while addressing skin tone gaps, are limited in diagnostic breadth and geographic diversity [24,25]. To strengthen fairness and reproducibility, skin tone annotation should evolve beyond the traditional Fitzpatrick phototypes toward newer scales such as the Monk Skin Tone scale, using standardized labeling and consistent documentation protocols across datasets [26].

Without datasets that reflect all segments of the population and systems that ensure access to recommended care, AI solutions may offer precise diagnoses that are ultimately inaccessible to those who need them most. Relying on single-modality datasets, such as static images alone, restricts the ability of AI systems to capture the real-world complexity of dermatological care [27]. Dermatologic decision-making is not merely visual; it involves psychometric, environmental, and cultural factors. For instance, 2 patients with the same lesion may receive different treatment recommendations depending on their medical history, stress levels, socioeconomic context, or cultural expectations.

**Figure 2.**
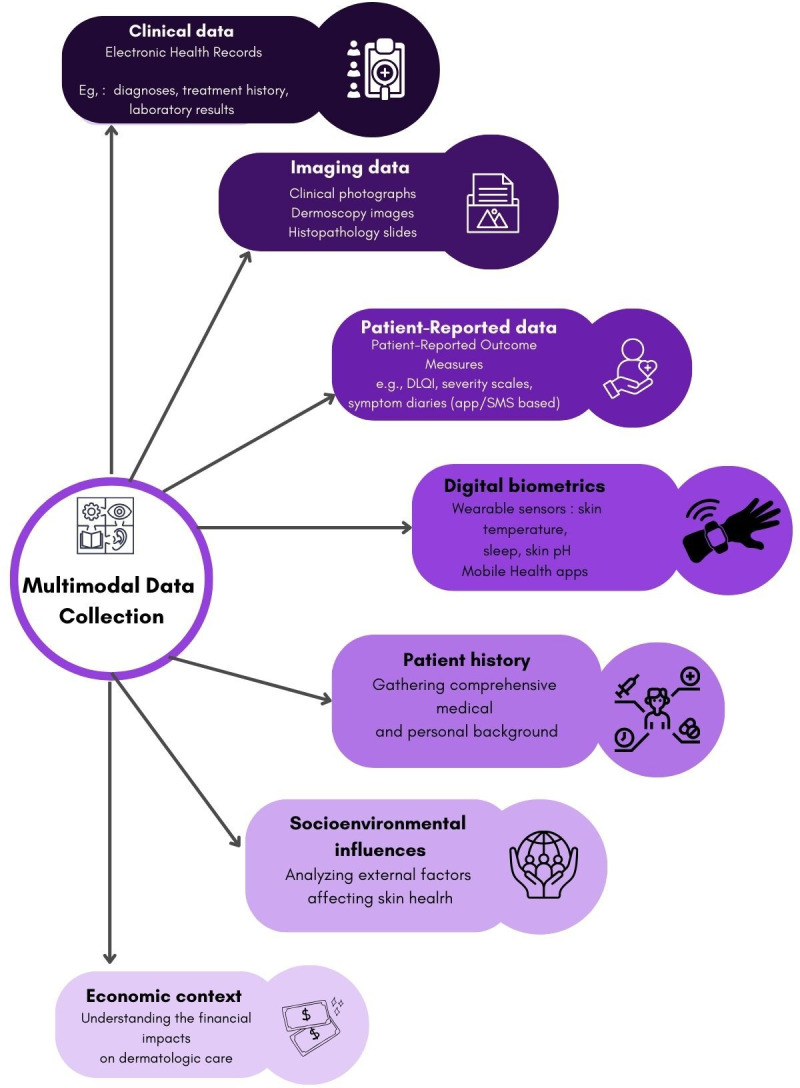
Expanding dermatology with multimodal data.

To address these issues, a multimodal data approach is essential (Figure 2) [28]. This includes integrating clinical photographs with patient histories, self-reported outcomes, environmental exposures, and behavioral data. For example, in conditions such as psoriasis or vitiligo, combining lesion photographs with validated severity indices (eg, Dermatology Life Quality Index and Pruritus Numerical Rating Scale), environmental parameters (UV index and humidity), comorbidities, and adherence telemetry can yield more personalized, context-aware interventions. These inputs can be captured via mHealth apps and wearables [29,30]. AI models trained on such holistic datasets, encompassing lesion images, lifestyle habits, stress levels, and treatment adherence, can deliver more accurate, individualized care. Importantly, these insights must be coupled with robust privacy safeguards, including on-device data processing and granular patient consent. Furthermore, to translate these innovations into improved outcomes in underserved areas, they must be linked to reliable access to medications through community health workers, local pharmacies, or public-sector distribution programs.

Enabling this shift requires interoperable and transparent data infrastructure with an emphasis on terminology mapping and handling of metadata. Platforms such as OpenMHealth, Apple HealthKit, and Fast Healthcare Interoperability Resources–compliant electronic health records can help structure and standardize multimodal data [31]. Future dermatology-specific tools must enable seamless integration of patient-reported outcomes and subjective (eg, pruritus scores) and objective (eg, lesion photos) metrics while ensuring data privacy. Ethical development and deployment of such AI tools must follow established frameworks such as FATE (fairness, accountability, transparency, and ethics) [32] and the OECD AI Principles [33]. These frameworks call for human oversight, robust transparency about model limitations, and equitable access to benefits. Poorly curated or biased data can not only reduce diagnostic accuracy but also worsen outcomes for already marginalized groups.

While appealing, the belief in autonomous, data-driven AI-based decision-making has significant limitations; data alone are not the answer. Although increasing data volume often enhances predictive accuracy, the quality and representativeness of the datasets remain crucial [34]. Poorly curated data can reinforce existing biases and, in some cases, worsen clinical outcomes [35]. Human clinicians inherently recognize that accurate interpretation of data requires understanding its context, particularly in dermatology, where the psychosocial impact of visible skin conditions demands sensitivity beyond numerical analysis alone [36]. Effective dermatological decision-making thus integrates objective data with patient-specific considerations, including emotional, social, and cultural factors. Identical skin lesions may necessitate different diagnostic or therapeutic approaches depending on individual patient circumstances and preferences, underscoring the necessity of collaboration between data-driven insights, clinical expertise, and patient perspectives.

## Combating Skin-Related Misinformation

Maximizing the impact of digital dermatology also requires confronting the parallel crisis of misinformation [37]. Fueled by algorithmic amplification and low digital health literacy, skin-related myths such as “sunscreen causes cancer” or “natural remedies are always safer” spread faster than verified medical guidance [38]. Platforms that reward engagement often privilege sensational content over accuracy, and dermatology is no exception. This dynamic is compounded by the low visibility of authoritative voices: only 4% of dermatology influencers on Instagram are board-certified dermatologists, and on TikTok, more than one-third of dermatology-related videos are created by nonprofessionals [39], frequently promoting unverified treatments such as raw potatoes for acne. On Reddit [40] and other forums, patients increasingly crowdsource diagnoses—sometimes for sensitive conditions such as sexually transmitted infections—reflecting growing distrust in formal health care and a shift toward peer-based digital health advice [41]. Studies of parenting blogs have shown that posts containing sunscreen misinformation consistently receive more engagement than scientifically accurate content [42].

Efforts to counter this phenomenon must go beyond reactive myth-busting and instead adopt structured, measurable, and trust-centered approaches and strategies grounded in successful digital literacy campaigns [43]. Public health initiatives in adjacent domains offer useful models. For example, the WHO’s “Pause Before You Share” campaign effectively encouraged users to reflect before forwarding unverified information during the COVID-19 pandemic [44]. The United Nations Children’s Fund and Gavi’s #VaccinesWork initiative combined localized messaging with influencer partnerships to rebuild vaccine confidence [45]. In sexual health, the AI-powered chatbot “Roo,” developed by Planned Parenthood, has demonstrated how conversational interfaces can deliver reliable, age-appropriate, and stigma-free health information at scale [46]. These campaigns share a common thread: they prioritize trust, accessibility, and proactive engagement, principles replicable in dermatology.

Drawing inspiration from these models, we propose an AI-powered dermatology chatbot (still in its conceptual phase) designed to address misinformation while empowering users with accurate, evidence-based content. The chatbot would operate on a retrieval-augmented generation architecture, fine-tuned on dermatology-specific content. It would draw from validated sources such as Cochrane systematic reviews, the American Academy of Dermatology (AAD), and the European Academy of Dermatology and Venereology (EADV) guidelines, patient association websites, and public health repositories from the WHO and CDC (Centers for Disease Control and Prevention). To ensure scientific rigor, the chatbot would internally rank information using the GRADE (Grading of Recommendations, Assessment, Development and Evaluations) framework. It would also include medical disclaimers, clinician escalation paths, hallucination checks, and refusal policies for diagnostic or treatment queries. Bias testing**,** data minimization**,** and content updates aligned with AAD and EADV guidelines would maintain fairness, privacy, and accuracy. Its performance should be tracked through reach, engagement, accuracy, harmful-advice rate, and user trust metrics. Dermatologists should lead content creation and partnerships to amplify credible, evidence-based information [47].

It is crucial to reiterate that dermatologists need to proactively develop accurate digital content to combat the spread of misinformation [48]. This can involve structured social media initiatives such as verified content series, myth-busting reels, or question-and-answer sessions on platforms where misinformation is most widespread. Collaborating with trusted influencers, patient advocacy groups, or public health campaigns can further expand reach and ensure that evidence-based information is both accessible and engaging [49].

## Beyond Content Curation: Developing a Technological Solution to Address Skin Misinformation

**Figure 3.**
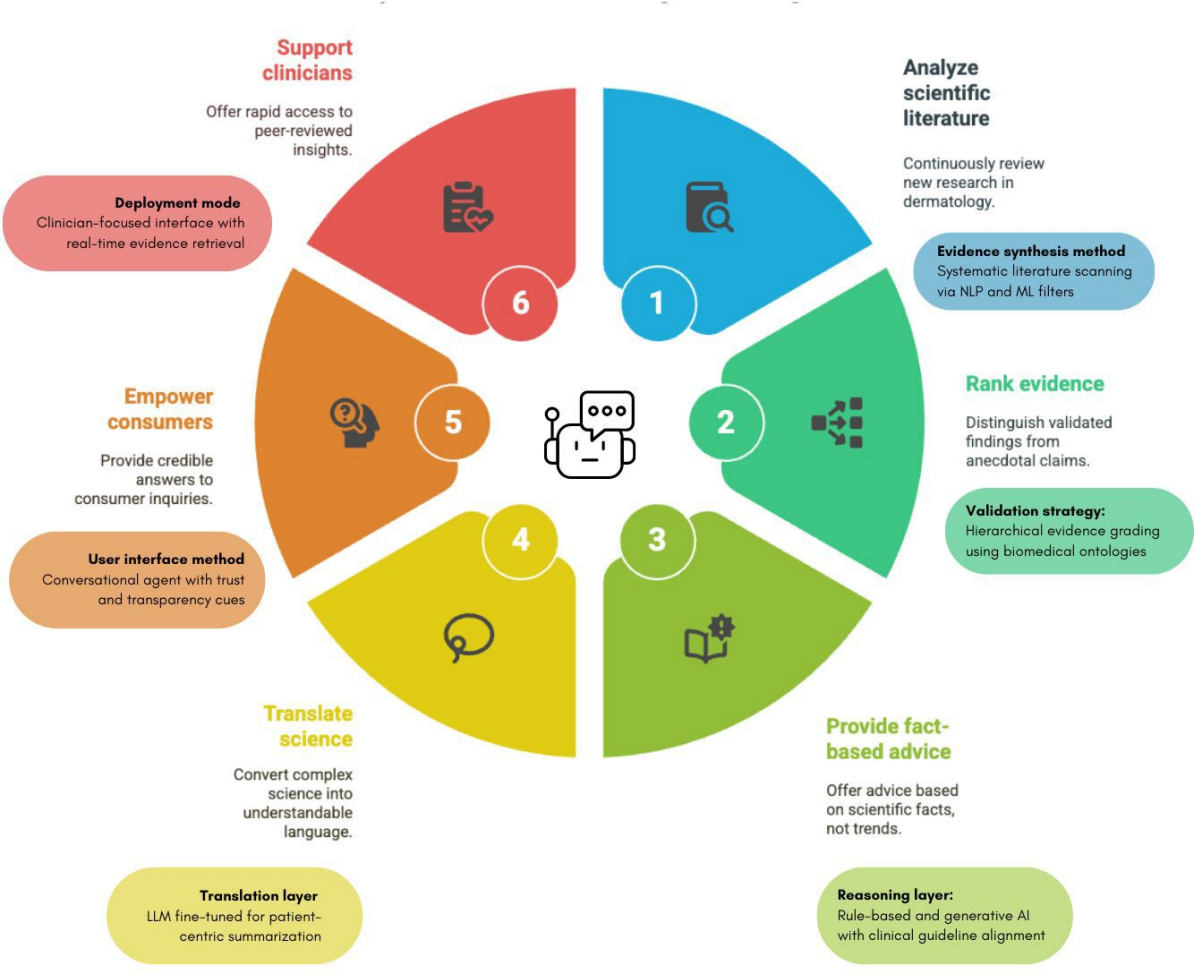
Cycle of artificial intelligence (AI)–powered dermatological chatbot. LLM: large language model; ML: machine learning; NLP: natural language processing.

The rapid, unchecked spread of skin-related misinformation is emerging as a significant public health concern—one that no dermatologist, no matter how dedicated, can tackle alone [48-51]. In this digital era, where falsehoods travel faster than facts, expert voices must be amplified by bold technological innovation. What is urgently needed is not just another fact sheet, but an intelligent, real-time solution: an AI-powered dermatology conversational agent (Figure 3) capable of delivering science-backed answers at the speed of the internet [52]. This chatbot could serve as a critical tool, helping to dispel myths, provide accurate information, and guide individuals toward appropriate care, with empathy [53].

Figure 3 outlines the engine behind such a tool—a dynamic cycle that analyzes scientific literature, ranks evidence, translates complex research into clear language, and delivers trustworthy, personalized guidance to both consumers and clinicians. This is not just an upgrade; it is a paradigm shift, and it demands a multidisciplinary alliance of dermatologists, AI developers, skin scientists, tech experts, and health communicators. Only together can we build the digital frontline dermatology desperately needs.

The concept of “radical dermatology” underscores this transformation. Radical dermatology refers to a future-oriented framework that calls on dermatologists and skin health stakeholders to actively lead, rather than passively follow, the digital transformation of their field. It recognizes the growing influence of big tech in reshaping skin health and emphasizes that dermatologists must drive this change through bold collaboration, clinical relevance, and one that is ethically governed through measures such as equity dashboards and data diversity thresholds.

While conceptual in origin, radical dermatology lays the groundwork for concrete steps such as pilot studies, comparative evaluations of AI- and clinician-led care, and assessments of cost-effectiveness and equity outcomes. However, limitations of this paper include its presentation of a conceptual framework and a hypothetical chatbot model, with insights derived exclusively from expert dialogue rather than empirical clinical trials. To advance this research, future work should aim at pilot studies across diverse populations, comparative analyses of AI-supported versus clinician-led care, and comprehensive cost-effectiveness research. Dermatologists must play a central role in leading this transformative shift, guiding not only the application of digital tools but also their fundamental design, rigorous testing, and governance. This crucial role requires dermatologists to do more than just use digital tools; they must actively shape the technological, ethical, and societal landscape of digital dermatology.

## Conclusions

Leveraging individual- and population-level skin data ethically through the convergence of computer vision, mHealth, and generative AI presents a pivotal opportunity for dermatology. Conversational tools such as dialogic AI or chatbots, when well designed, can interpret validated evidence, provide real-time responses, and deliver guidance in ways that are accessible, accurate, and empathetic [52,53]. This is particularly relevant in dermatology, where care is visual, is time sensitive, and depends heavily on patient education and reassurance.

The promise of digital dermatology will only be achieved if these tools are developed for everyone, with transparency, accuracy, and trust as core principles and with an emphasis on ongoing safety auditing. People without access to dermatology care or reliable information stand to gain the most, provided their needs are addressed from the outset. Deployment should therefore prioritize underserved users through multilingual interfaces, offline functionality, and low-bandwidth design as integral components of equity-centered implementation. Dermatologists must remain central to this process, guiding design, overseeing implementation, and setting ethical standards to ensure that digital solutions are clinically sound and culturally appropriate.

The future of dermatology will be shaped not by algorithms alone but through collaboration among clinicians, technologist researchers, policymakers, and patients to ensure technology meaningfully advances skin health for all.

## Abbreviations

AAD: American Academy of Dermatology
AI: artificial intelligence
CDC: Centers for Disease Control and Prevention
EADV: European Academy of Dermatology and Venereology
FATE: fairness, accountability, transparency, and ethics
GRADE: Grading of Recommendations, Assessment, Development and Evaluations
mHealth: mobile health
WHO: World Health Organization

## References

[1] Szeto MD, Alhanshali L, Rundle CW (2024). Dermatologic data from the global burden of disease study 2019 and the patientslikeme online support community: comparative analysis. JMIR Dermatol.

[2] Wehausen B, Hill DE, Feldman SR (2016). Most people with psoriasis or rosacea are not being treated: a large population study. Dermatol Online J.

[3] Yakupu A, Aimaier R, Yuan B (2023). The burden of skin and subcutaneous diseases: findings from the global burden of disease study 2019. Front Public Health.

[4] (2025). Skin diseases as a global public health priority. World Health Organization.

[5] Skin and Digital Summit.

[6] International Masters Course on Aging Science (IMCAS).

[7] du Crest D, Madhumita M, Enbiale W (2024). Skin and digital-the 2024 narrative. Mayo Clin Proc Digit Health.

[8] Adepoju O, Dang P, Nguyen H, Mertz J (2024). Equity in digital health: assessing access and utilization of remote patient monitoring, medical apps, and wearables in underserved communities. Inquiry.

[9] Gwillim EC, Azzawi S, Aigen AR (2024). Underserved populations and health equity in dermatology: digital medicine and the role of artificial intelligence. Clin Dermatol.

[10] Schaekermann M, Spitz T, Pyles M (2024). Health equity assessment of machine learning performance (HEAL): a framework and dermatology AI model case study. EClinicalMedicine.

[11] Ahuja S, Briggs SM, Collier SM (2022). Teledermatology in rural, underserved, and isolated environments: a review. Curr Dermatol Rep.

[12] Chang AY, Kiprono SK, Maurer TA (2017). Providing dermatological care in resource-limited settings: barriers and potential solutions. Br J Dermatol.

[13] Wongvibulsin S, Yan MJ, Pahalyants V, Murphy W, Daneshjou R, Rotemberg V (2024). Current state of dermatology mobile applications with artificial intelligence features. JAMA Dermatol.

[14] Sun MD, Kentley J, Mehta P, Dusza S, Halpern AC, Rotemberg V (2022). Accuracy of commercially available smartphone applications for the detection of melanoma. Br J Dermatol.

[15] Alami H, Rivard L, Lehoux P (2020). Artificial intelligence in health care: laying the foundation for responsible, sustainable, and inclusive innovation in low- and middle-income countries. Global Health.

[16] (2024). The WHO Skin NTDs app shows encouraging results in Kenya study. World Health Organization.

[17] Richardson S, Lawrence K, Schoenthaler AM, Mann D (2022). A framework for digital health equity. NPJ Digit Med.

[18] Sylla B, Ismaila O, Diallo G (2025). 25 years of digital health toward universal health coverage in low- and middle-income countries: rapid systematic review. J Med Internet Res.

[19] Duniphin DD (2023). Limited access to dermatology specialty care: barriers and teledermatology. Dermatol Pract Concept.

[20] Orton M, Agarwal S, Muhoza P, Vasudevan L, Vu A (2018). Strengthening delivery of health services using digital devices. Glob Health Sci Pract.

[21] Pinto-Coelho L (2023). How artificial intelligence is shaping medical imaging technology: a survey of innovations and applications. Bioengineering (Basel).

[22] Daneshjou R, Vodrahalli K, Novoa RA (2022). Disparities in dermatology AI performance on a diverse, curated clinical image set. Sci Adv.

[23] Narla A, Kuprel B, Sarin K, Novoa R, Ko J (2018). Automated classification of skin lesions: from pixels to practice. J Invest Dermatol.

[24] Groh M, Harris C, Daneshjou R, Badri O, Koochek A (2022). Towards transparency in dermatology image datasets with skin tone annotations by experts, crowds, and an algorithm. Proc ACM Hum-Comput Interact.

[25] Jain T (2024). Evaluating machine learning-based skin cancer diagnosis. arXiv.

[26] Arora A, Alderman JE, Palmer J (2023). The value of standards for health datasets in artificial intelligence-based applications. Nat Med.

[27] Mishra S, Chaudhury S, Imaizumi H, Yamasaki T (2020). Assessing robustness of deep learning methods in dermatological workflow. arXiv.

[28] Krones F, Marikkar U, Parsons G, Szmul A, Mahdi A (2025). Review of multimodal machine learning approaches in healthcare. Information Fusion.

[29] Hong J, Mosca M, Hadeler E, Hakimi M, Bhutani T, Liao W (2021). The future of personalized medicine in psoriasis. Dermatological Reviews.

[30] Ahuja R, Narayanan B, Gupta S (2022). Precision medicine and personalized approach in vitiligo. Dermatological Reviews.

[31] Gordon WJ, Landman A, Zhang H, Bates DW (2020). Beyond validation: getting health apps into clinical practice. NPJ Digit Med.

[32] Singhal A, Neveditsin N, Tanveer H, Mago V (2024). Toward fairness, accountability, transparency, and ethics in ai for social media and health care: scoping review. JMIR Med Inform.

[33] Yeung K (2020). Recommendation of the Council on Artificial Intelligence (OECD). Int leg mater.

[34] Okolo CT (2022). Optimizing human-centered AI for healthcare in the Global South. Patterns (N Y).

[35] Green BL, Murphy A, Robinson E (2024). Accelerating health disparities research with artificial intelligence. Front Digit Health.

[36] Breen N, Berrigan D, Jackson JS (2019). Translational health disparities research in a data-rich world. Health Equity.

[37] Nelson EE, Black TA, Rousseau MA, Rashid RM (2023). Combating misinformation in dermatology. Dermatol Pract Concept.

[38] Ecker U, Roozenbeek J, van der Linden S (2024). Misinformation poses a bigger threat to democracy than you might think. Nature New Biol.

[39] Ranpariya V, Chu B, Fathy R, Lipoff JB (2020). Dermatology without dermatologists? Analyzing Instagram influencers with dermatology-related hashtags. J Am Acad Dermatol.

[40] Chu B, Fathy R, Nobles AL, Lipoff JB (2021). Patient crowdsourcing of dermatologic consults on a Reddit social media community. J Am Acad Dermatol.

[41] Nobles AL, Leas EC, Althouse BM (2019). Requests for diagnoses of sexually transmitted diseases on a social media platform. JAMA.

[42] O’Connor C, Rafferty S, Murphy M (2022). A qualitative review of misinformation and conspiracy theories in skin cancer. Clin Exp Dermatol.

[43] Sood A, Sangari A, Stoff BK (2023). Navigating internet-based misinformation with patients in the clinic. J Am Acad Dermatol.

[44] Rodrigo P, Arakpogun EO, Vu MC, Olan F, Djafarova E (2022). Can you be mindful? The effectiveness of mindfulness-driven interventions in enhancing the digital resilience to fake news on COVID-19. Inf Syst Front.

[45] Germani F, Biller-Andorno N (2022). How to counter the anti-vaccine rhetoric: filling information voids and building resilience. Hum Vaccin Immunother.

[46] Mills R, Mangone ER, Lesh N, Jayal G, Mohan D, Baraitser P (2024). Chatbots that deliver contraceptive support: systematic review. J Med Internet Res.

[47] Atkins D, Best D, Briss PA (2004). Grading quality of evidence and strength of recommendations. BMJ.

[48] Szeto MD, Mamo A, Afrin A, Militello M, Barber C (2021). Social media in dermatology and an overview of popular social media platforms. Curr Dermatol Rep.

[49] El Mikati IK, Hoteit R, Harb T (2023). Defining misinformation and related terms in health-related literature: scoping review. J Med Internet Res.

[50] Mehta-Ambalal SR, Nisarta M (2021). Dermatology 2.0- how the internet is changing us, our patients and our practice. Indian Dermatol Online J.

[51] O’Connor C, O’Grady C, Murphy M (2022). Spotting fake news: a qualitative review of misinformation and conspiracy theories in acne vulgaris. Clin Exp Dermatol.

[52] Mariani MM, Hashemi N, Wirtz J (2023). Artificial intelligence empowered conversational agents: a systematic literature review and research agenda. J Bus Res.

[53] Chen D, Chauhan K, Parsa R (2025). Author correction: patient perceptions of empathy in physician and artificial intelligence chatbot responses to patient questions about cancer. NPJ Digit Med.

